# Asymptomatic leishmaniasis in a patient with relapsed multiple myeloma

**DOI:** 10.1002/jha2.217

**Published:** 2021-05-13

**Authors:** Lucia Rubio, Maria Jimenez

**Affiliations:** ^1^ Department of Hematology Hospital Universitario Doctor Peset Valencia Spain


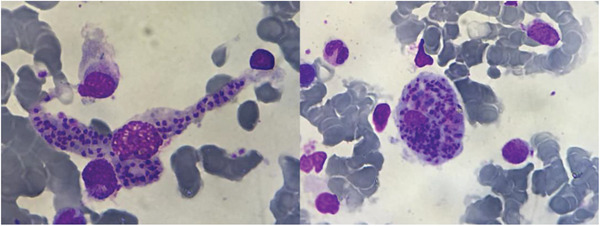


A 52‐year‐old man with IgG lambda, ISS 3 multiple myeloma (MM) in first relapse, was receiving treatment with isatuximab, carfilzomib, and dexamethasone as part of a clinical trial. Myeloma restaging after 15 cycles of therapy showed an unremarkable complete blood count. Following protocol procedures, a bone marrow aspirate was performed as part of the MM evaluation. Examination of the bone marrow aspirate revealed amastigotes (image A and B, × 100 objective), the typical appearance of leishmaniasis, and Giemsa stain as well as polymerase‐chain‐reaction testing of the bone marrow aspirate confirmed the diagnosis. The patient was asymptomatic, the physical examination revealed no organ enlargements or skin alterations. Abdominal ultrasound detected the presence of a mildly enlarged spleen with a legth of 14.5 cm. Transmitted by sandflies, *L. infantum* is endemic in the Mediterranean region and remains a serious parasitic disease, causing high morbidity and mortality in the developing world. The patient completed treatment with liposomal amphotericin B and remains asymptomatic, with no evidence of leishmaniasis in the bone marrow and normal laboratory values. Hematologists should be alert to an incidental diagnosis that can have a marked impact on patient management.

